# A comprehensive study of phospholipid fatty acid rearrangements in metabolic syndrome: correlations with organ dysfunction

**DOI:** 10.1242/dmm.043927

**Published:** 2020-06-17

**Authors:** Amélie Bacle, Linette Kadri, Spiro Khoury, Romain Ferru-Clément, Jean-François Faivre, Christian Cognard, Jocelyn Bescond, Amandine Krzesiak, Hugo Contzler, Nathalie Delpech, Jenny Colas, Clarisse Vandebrouck, Stéphane Sébille, Thierry Ferreira

**Affiliations:** 1 Laboratoire “Lipotoxicity and Channelopathies (LitCh) - ConicMeds”, Université de Poitiers, 1, rue Georges Bonnet, 86073 Poitiers, France; 2 Laboratoire “Signalisation et Transports Ioniques Membranaires (STIM; EA 7349)”, Université de Poitiers, 1, rue Georges Bonnet, 86073 Poitiers, France; 3 Laboratoire “Mobilité Vieillissement et Exercice (MOVE; EA 6314)”, Université de Poitiers, 8, Allée Jean Monnet, 86073 Poitiers, France

**Keywords:** Saturated fat, Phospholipids, Type 2 diabetes, Cardiovascular disease, Hepatic steatosis, Polyunsaturated fatty acids

## Abstract

The balance within phospholipids (PLs) between saturated fatty acids and monounsaturated or polyunsaturated fatty acids is known to regulate the biophysical properties of cellular membranes. As a consequence, in many cell types, perturbing this balance alters crucial cellular processes, such as vesicular budding and the trafficking/function of membrane-anchored proteins. The worldwide spread of the Western diet, which is highly enriched in saturated fats, has been clearly correlated with the emergence of a complex syndrome known as metabolic syndrome (MetS). MetS is defined as a cluster of risk factors for cardiovascular diseases, type 2 diabetes and hepatic steatosis; however, no clear correlations have been established between diet-induced fatty acid redistribution within cellular PLs and the severity/chronology of the symptoms associated with MetS or the function of the targeted organs. To address this issue, in this study we analyzed PL remodeling in rats exposed to a high-fat/high-fructose diet (HFHF) over a 15-week period. PL remodeling was analyzed in several organs, including known MetS targets. We show that fatty acids from the diet can redistribute within PLs in a very selective manner, with phosphatidylcholine being the preferred sink for this redistribution. Moreover, in the HFHF rat model, most organs are protected from this redistribution, at least during the early onset of MetS, at the expense of the liver and skeletal muscles. Interestingly, such a redistribution correlates with clear-cut alterations in the function of these organs.

This article has an associated First Person interview with the first author of the paper.

## Abstract (precis)

**Summary:** In rats, a high-fat diet induces the selective distribution of fatty acids within phosphatidylcholine in the liver and muscles, in a manner that correlates with organ dysfunction. The cardiovascular system appears to be protected under these conditions.

## INTRODUCTION

Initial observations of the involvement of obesity and dyslipidemia in the occurrence of metabolic disorders, such as type 2 diabetes, fatty liver and cardiovascular diseases, go back to the late 1960s. Since then, the prevalence of this metabolic syndrome has been clearly correlated with the worldwide spread of the Western diet, which is excessively rich in sugar and saturated fat. In obese individuals, the incidence of metabolic syndrome is associated with high plasma levels of non-esterified fatty acids (NEFAs) and, more specifically, of long-chain saturated fatty acids (SFAs) (Kusminski et al., 2009). A prime example of a long-chain SFA is palmitate (comprising 16 carbon atoms and no double-bond in the acyl chain, 16:0), the main component of palm oil. When the storage capacity of the adipose tissue is exceeded, NEFAs tend to accumulate in cells not suited for lipid storage, among which muscle cells, hepatocytes and pancreatic β-cells are prime examples (Kusminski et al., 2009). As a corollary, it is now widely accepted that fatty acid imbalances are directly involved in the promotion of insulin resistance, non-alcoholic steatohepatitis, impaired glucose tolerance and systemic inflammation (Cascio et al., 2012).

Phospholipids (PLs), which contain two fatty acid chains, are the main components of cellular membranes. In mammalian cells, phosphatidylcholine (PC) is the most abundant PL (Christie, 1985). Ethanolamine (Etn)-containing PL species are the second most abundant PLs, of which phosphatidylethanolamine (PE), a diacyl glycerophospholipid, and ethanolamine plasmalogen [PE(P)], an alkenylacylglycerophospholipid, are the main constituents (Braverman and Moser, 2012; Honsho and Fujiki, 2017). These species, and to a lesser extent phosphatidylinositol (PI) and phosphatidylserine (PS), are the most abundant lipid classes whatever the organ considered; for example, they constitute 75% of all lipid species in the heart of rats and 79% in the liver (Christie, 1985). Maintaining the equilibrium between SFAs, monounsaturated and polyunsaturated fatty acids (UFAs) within membrane PLs is crucial to sustain the optimal membrane biophysical properties compatible with selective organelle-based processes, including vesicular budding or membrane-protein trafficking and function (Antonny et al., 2015). As a corollary, impaired balances within SFAs and UFAs have been shown to result in dramatic cellular dysfunction in cells relevant to metabolic syndrome (for a review, see Bacle and Ferreira, 2019). For example, altered insulin secretion in pancreatic β-cells or impaired glucose disposal in muscle and liver cells have been reported in response to SFA overload (Bacle and Ferreira, 2019). This process, which is referred to as lipotoxicity, ultimately leads to cell death by apoptosis in all the cellular systems tested (Bacle and Ferreira, 2019).

Many animal models of metabolic syndrome, either genetic or diet-induced, have been described (Wong et al., 2016). Recently, Lozano et al. (2016) reported an elegant rat model based on a high-fructose and high-fat diet (HFHF). These authors demonstrated that the combination of high fat and high carbohydrate induced type 2 diabetes with widespread tissue effects. The phenotype increased gradually from 2 to 8 months following the shift to HFHF, with insulin resistance and hepatic disorders increasing progressively to reach a maximum at this latter time point. Because of the increased consumption of sugar-rich and fatty products, and an increase in preference for such products, metabolic disorders are becoming more common at a younger age in humans. Thus, this model seems very appealing to evaluate the chronology of the effects of the diet, particularly during the early onset of metabolic syndrome.

In this context, the present study aimed to evaluate the distribution of fatty acids coming from the diet within cellular PL within various organs in the HFHF model. Moreover, the function of those organs most affected by fatty acid distribution was also evaluated.

## RESULTS

### Metabolic follow-up

**Fig. 1.**
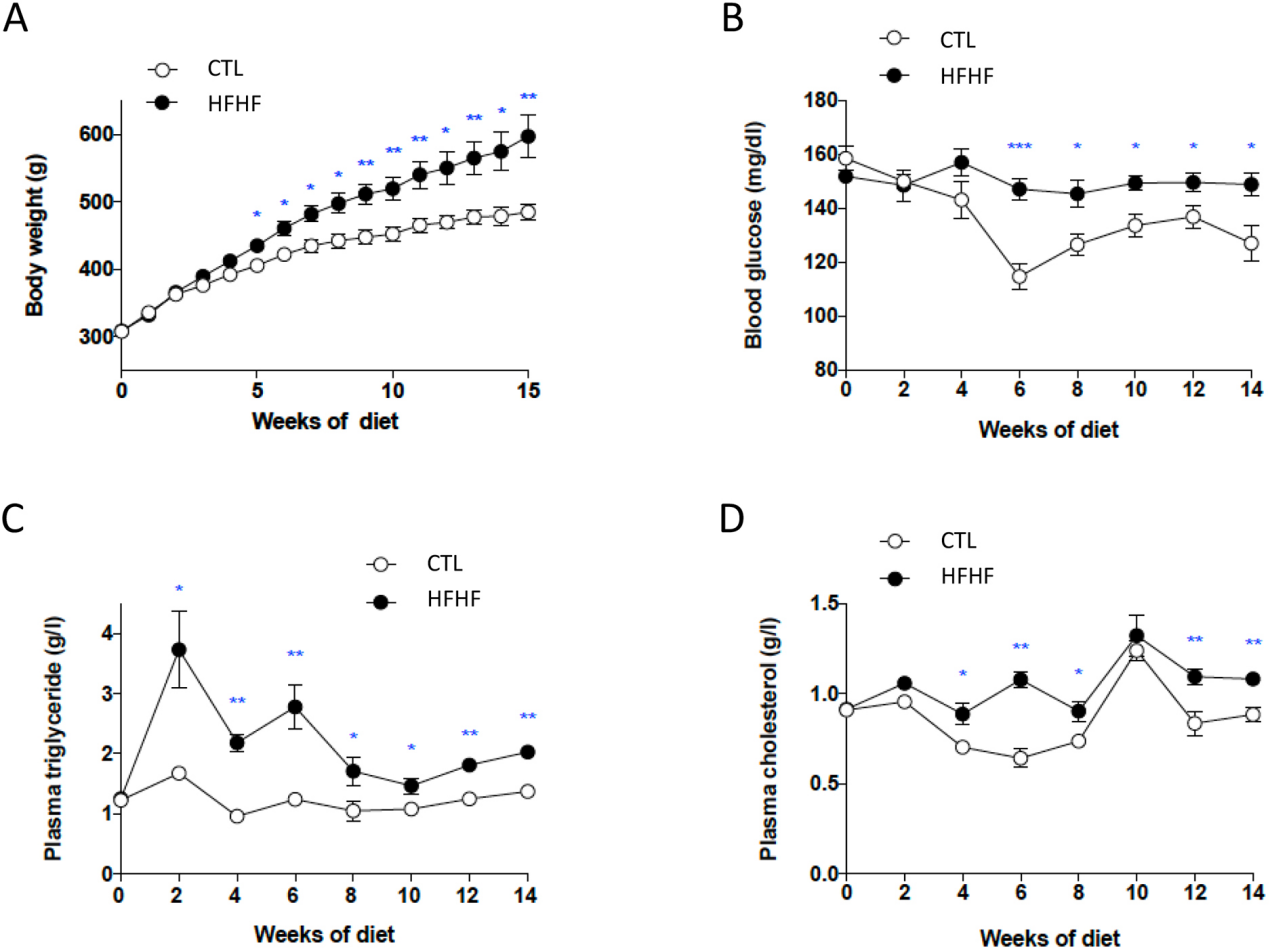
**Longitudinal measurements (under random fed conditions) performed during the study.** (A-D) During the longitudinal observation, body weight was measured each week (A) and plasma glucose (B), plasma triglycerides (C) and plasma cholesterol (D) levels were determined every 2 weeks at the same time of day (14:00) for CTL (*n*=8) and HFHF rats (*n*=8). Protocols used are described in the Materials and Methods section. Data are presented as means±s.d. Parameters were compared between CTL and HFHF rats using unpaired Student’s *t*-test; ***P<0.001, **P<0.01 and *P<0.05.

After 5 weeks of diet, HFHF induced a significant increase in body weight (*P*<0.05) maintained until the end of the study, in comparison with the standard control diet (CTL; Fig. 1A). Alterations in glucose homeostasis were visible from the sixth week under HFHF (Fig. 1B). The HFHF rats also developed dyslipidemia, with a significant increase in blood triglyceride and cholesterol levels as early as 2 and 4 weeks of age, respectively (Fig. 1C,D). These data recapitulated the observations from Lozano et al. (2016). At 15 weeks, additional metabolic measurements were performed on 12 rats after prior gastric emptying (six CTL and six HFHF; see below); all animals were sacrificed to perform the PL analyses and *ex vivo* experiments described below.

### Fatty acid distribution within PLs varies depending on the organ

**Fig. 2.**
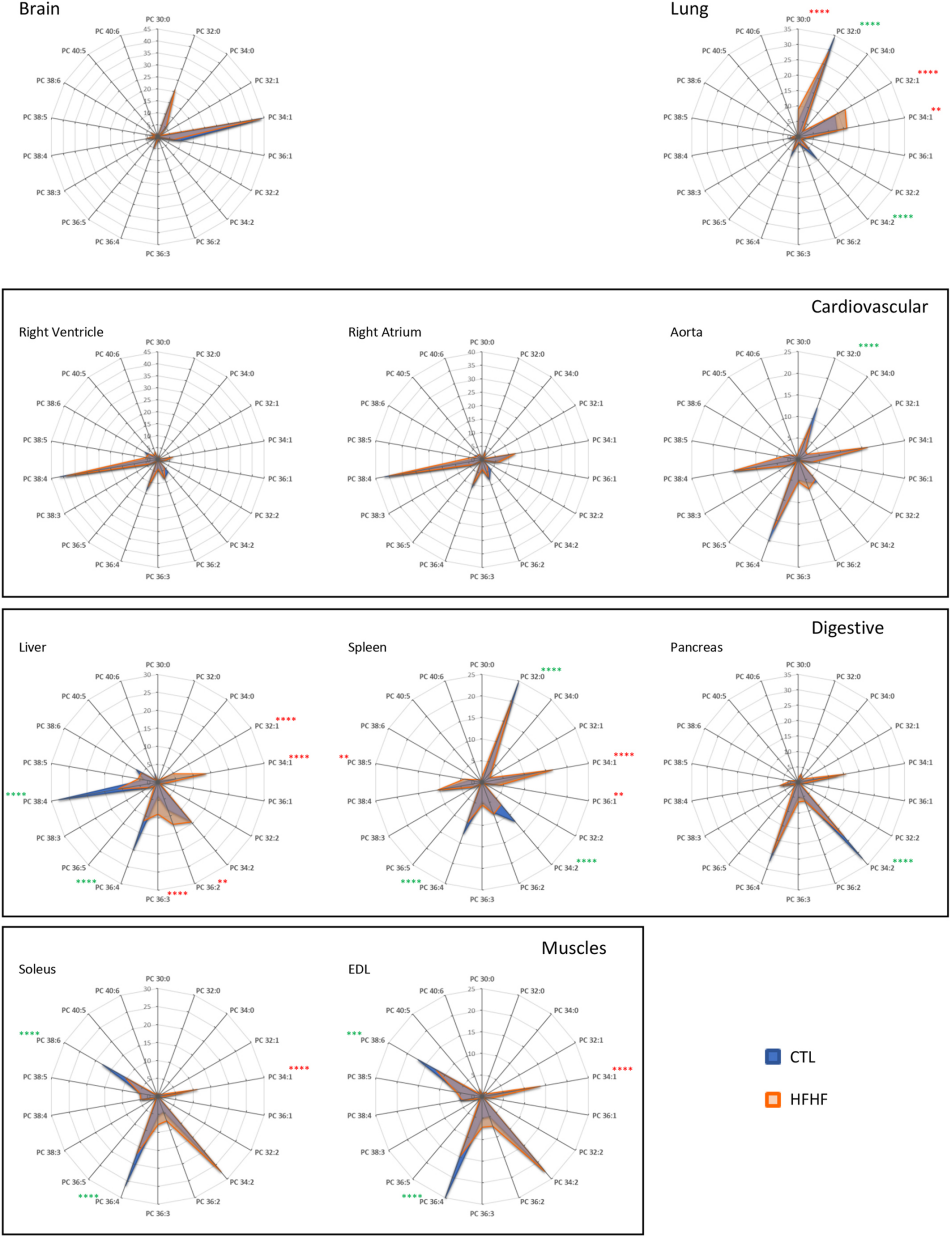
**PC species distribution in various organs as a function of the diet.** Total lipids were extracted and phospholipid species were purified and analyzed by ESI-MS from samples corresponding to the indicated organs obtained from rats fed either with a control (CTL) or HFHF diet, as described in the Materials and Methods section. PC subspecies distribution is shown in each case. The total carbon chain length (x) and number of carbon-carbon double bonds (y) of the main PC molecular species (x:y) are indicated. Values are the mean±s.d. of four independent determinations from four individuals from both groups in each case. Statistical analysis was performed using two-way ANOVA and completed by Bonferroni post-tests to compare means variation between the two groups of animals for each PC subspecies. Significant differences between CTL and HFHF are indicated (****P<0.0001, ***P<0.001 and **P<0.01) either in green, if a specific subspecies is decreased under the HFHF diet as compared with CTL, or in red, if this subspecies is increased under the HFHF regimen.

In a first step, we determined the fatty acid distribution within the different PL species, namely PC, PE, PS and PI in various organs from rats on a standard diet (CTL). This study was completed by the analysis of plasmalogens, which correspond to specific glycerophospholipid species containing a vinyl-ether bond at the *sn-*1 position (Braverman and Moser, 2012). Among this lipid class, ethanolamine PE(P)s are found in all rat organs (Braverman and Moser, 2012; see below), whereas choline plasmalogens, which can be found in significant amounts in specific human tissues, are only detected in trace amounts (Braverman and Moser, 2012; S.K. and T.F., unpublished data). The present study was performed on known targets of metabolic syndrome-induced disorders, such as the liver, skeletal muscles (extensor digitorum longus and soleus muscles), the vascular system (heart and aorta) and the pancreas, and was complemented by the same analyses on the brain, spleen and lung. With this aim, total PLs were extracted from the various organs and analyzed by mass spectrometry, in both the positive- and negative-ion modes, as described in the Materials and Methods section. Examples of characteristic spectra obtained in the negative- and positive-ion modes are shown in Figs S1 and S2, respectively. The results obtained for PC are displayed in Fig. 2 as radar graphs and Fig. S3 as histograms; the data corresponding to PI, PE, PS and PE(P) are shown in Figs S4-S7. In these figures, PL species are denominated by their initials followed by the total number of carbons and the number of carbon-carbon double bonds in their acyl chains (e.g. PC 38:4 corresponds to a PC containing 38 carbon atoms and four double bonds in its acyl chains).

As described in previous studies (Hicks et al., 2006), a first observation is that PC is the PL species that displays the widest variations in terms of fatty acid chain distribution depending on the organ considered (Fig. 2 and Fig. S3), whereas PI essentially appears as a major species (PI 38:4) in all the organs studied (Fig. S4).

Concerning PC, it clearly appears that some organs are particularly enriched in species bearing polyunsaturated fatty acid chains (PUFAs; more than two double bonds/unsaturations in their fatty acid chains, e.g. PC 38:4), whereas others preferentially contain PC with two saturated fatty acyl chains (e.g. PC 32:0) (Fig. 2).

**Fig. 3.**
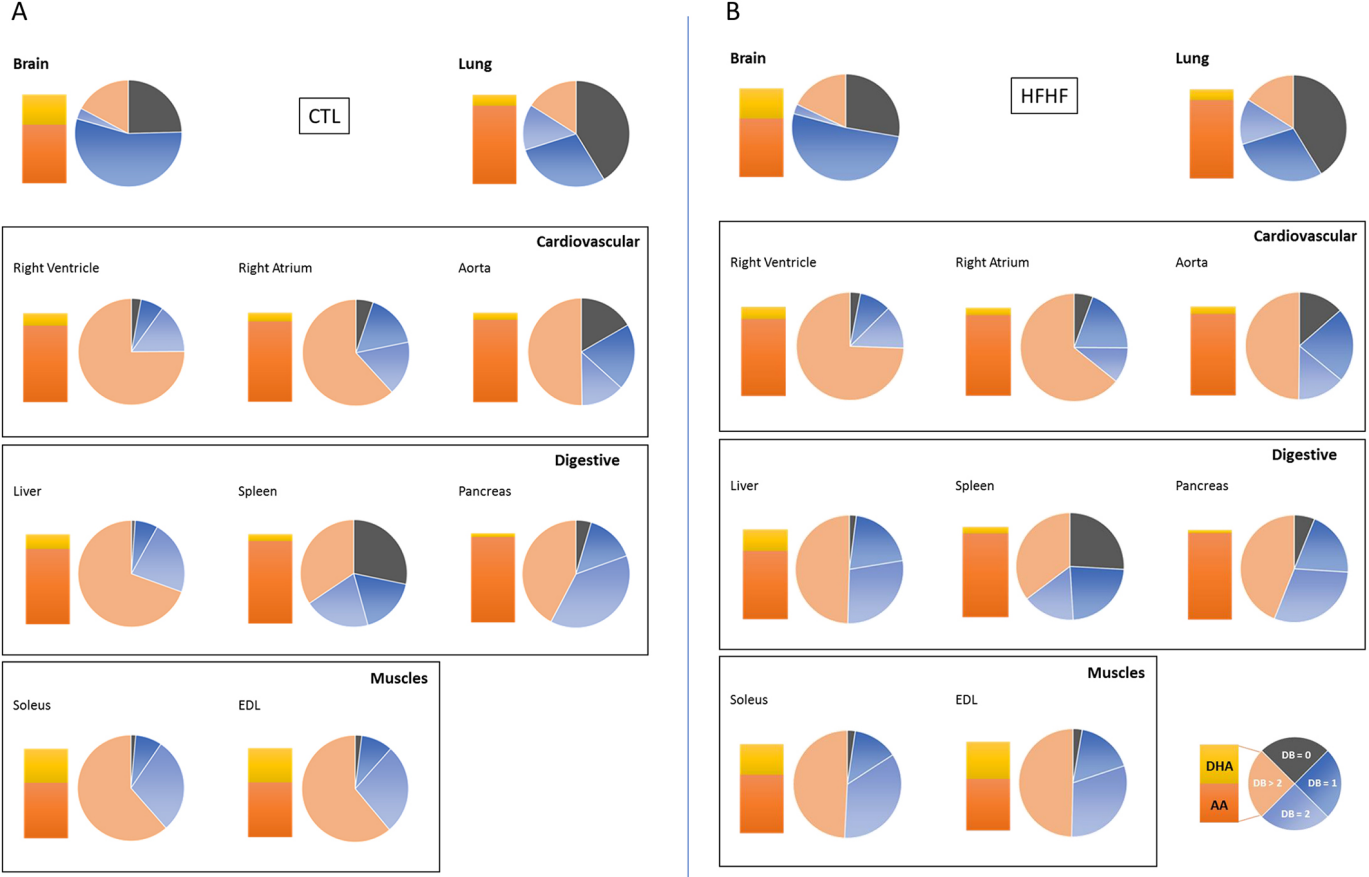
**PC double-bond (DB) index and DHA to AA ratios in various organs as a function of the diet.** (A,B) Total lipids were extracted and phospholipid species were purified and analyzed by ESI-MS from samples corresponding to the indicated organs obtained either from rats fed with a normal CTL diet (A) or HFHF diet (B), as described in the Materials and Methods section. The relative percentage of saturated (DB=0, no double bonds) versus monounsaturated (DB=1, one double bond), diunsaturated (DB=2, two double bonds) and polyunsaturated (DB>2, more than two double bonds) PC species was obtained from the PC subspecies distribution displayed in Fig. 2. The ratio of DHA- to AA-containing PC subspecies in the various organs is also displayed.

**Fig. 4.**
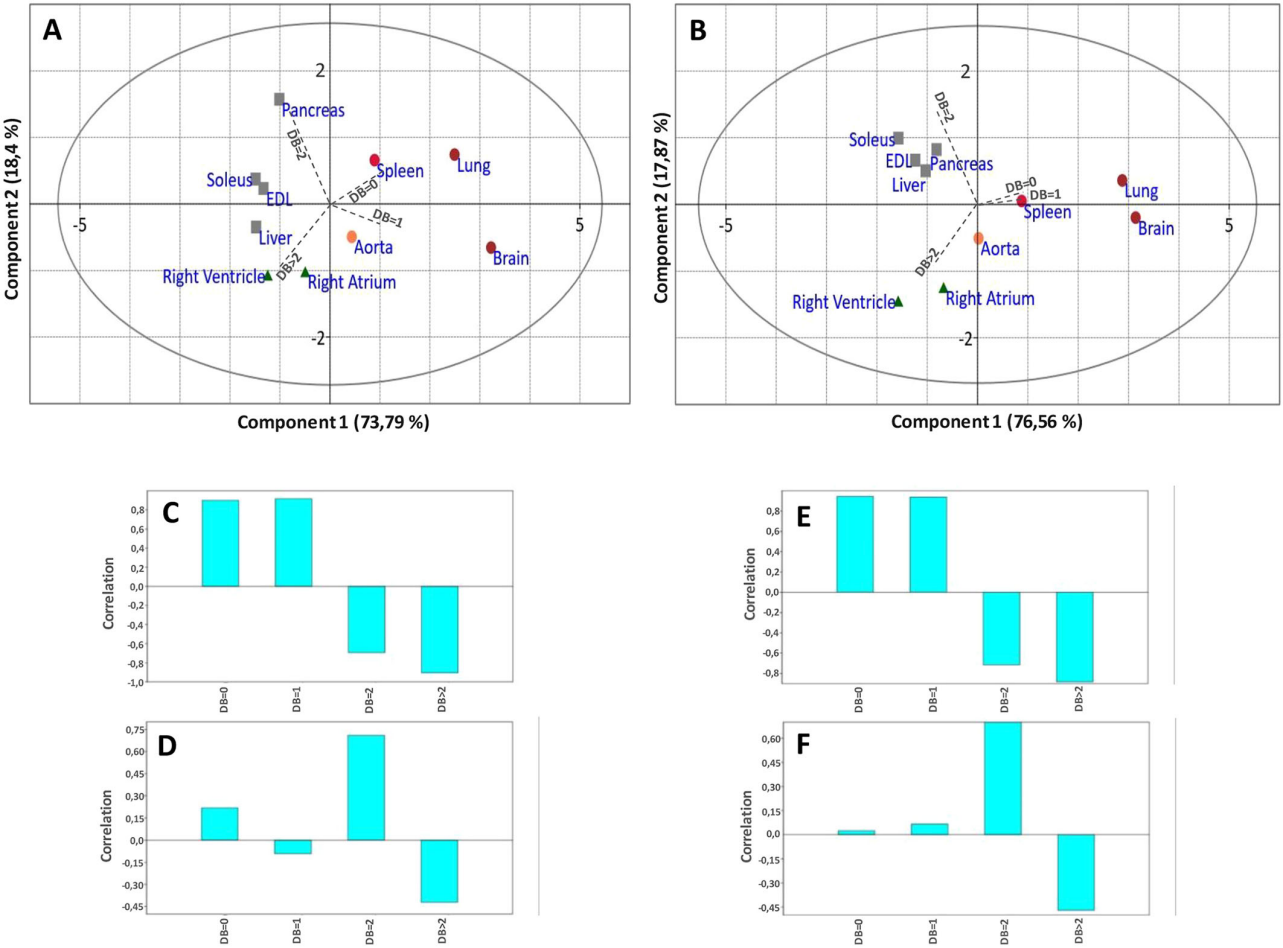
**PCAs of the PC DB index as a function of the organ and in response to the HFHF diet.** (A,B) PCA score plot of the different organs in the normal (CTL) diet (A) and in the HFHF diet (B). (C,D) PCA-1 (C) and PCA-2 (D) loading plots for A. (E,F) PCA-1 (E) and PCA-2 (F) loading plots for B. DB is the number of double bonds in PC species.

To better visualize these variations, the double-bond (DB) index was calculated in each case (Fig. 3 and Figs S8-S11). The DB index was calculated by considering the total number of double bonds in both acyl chains of each PC species: for example, DB=0 was obtained by summing up the percentage of all the PC species bearing two saturated fatty acid chains, that is zero double bonds in their acyl chains (e.g. PC 30:0; PC32:0…). When performing a multivariate principal component analysis (PCA) based on the DB index (Fig. 4A,C,D), the striking differences between the various organs could be clearly visualized. The projection of the studied organs on component 1 and component 2 in the score plot shows that these organs can be classified according to this index. As shown, the liver, skeletal muscles and the cardiovascular system are particularly enriched in PUFA-containing PC species (DB>2). By contrast, the spleen, the brain and the lung contain remarkably high amounts of saturated PC species (DB=0). The brain also differs from other organs in its high levels of monounsaturated PC species (DB=1). In the latter case, PC 34:1 appears as the major species. The pancreas also displays a very characteristic signature, with high levels of diunsaturated PC species (DB=2).

Among PUFAs, important variations could also be observed depending on the organ considered (Figs 2 and 3A). If arachidonic acid (AA; 20:4) appeared as the most represented fatty acid in pancreas, spleen, liver, lung and the cardiovascular system, as combinations with palmitate (PC 36:4) or stearate (PC 38:4; Fig. 2 and Table 1), docosahexaenoic acid (DHA; 22:6) was exquisitely enriched in skeletal muscles, in combination with palmitate (PC 38:6; Fig. 2 and Table 1).

**Table 1.**
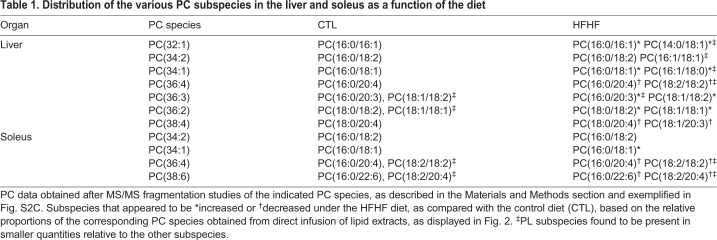
**Distribution of the various PC subspecies in the liver and soleus as a function of the diet**

PE differed from PC in the sense that PUFA-containing species were systematically dominant, whatever the organ considered (Figs S5 and S9A); however, the DHA to AA balance greatly varied among organs. As already observed for PC, DHA was particularly enriched in skeletal muscles where PE 40:6 was the most common species. Interestingly, DHA was also the most prominent PUFA in the brain, an organ displaying very different PC behavior (Figs S2 and S3A). In the heart, although very similar PC signatures were observed for the ventricles and atria, PE 40:6 was enriched in the ventricles as compared with other organs. Although less marked than for PC, the brain, spleen and lung appeared as the organs containing the highest amounts of saturated PE and the pancreas as the organ most enriched with DB=2 species.

The fatty acid distribution within PS paralleled that observed with PE (Figs S6 and S10A), with PUFA being the major fatty acids in all organs considered. Again, DHA was the most represented PUFA in skeletal muscles, brain, ventricle and atrium (PS 40:6), whereas AA was the most abundant in other organs (PS 38:4). The pancreas differed from others in a wider distribution of the fatty acyl chains and relatively high levels of DB=2 species.

As described elsewhere (Braverman and Moser, 2012), PE(P)s were detected in all of the organs analyzed in this study, where they comprised between 30% and 56% of ethanolamine-containing glycerophospholipids [i.e. PE and PE(P)]. The exception to this was the liver, in which PE(P) levels dropped to only 7% (data not shown). Whatever the organ considered, PUFA-containing species were the most prominent (Figs S7 and S11A) with, as for PE and PS, a selective enrichment in DHA in the brain, the ventricles and skeletal muscles.

To summarize, all the organs studied here displayed very characteristic fatty acid distributions within PLs and, therefore, various saturation rates: the organs can be classified as DB=0/saturated organs (spleen, lung), DB=1 (brain), DB=2 (pancreas) and DB>2 organs (liver, muscle and cardiovascular system). Among the latter, one can differentiate AA (liver and cardiovascular system)- and DHA (skeletal muscle)-enriched organs. The brain is the organ that displays the most discrepancies between PLs. PC is predominantly DB=1, whereas PE and PS are essentially DHA-enriched. These observations match and complete previous observations made by others (Hicks et al., 2006).

### PL species are differently affected by the HFHF diet, in an organ-specific manner

The high-fat diet used in this study is exquisitely enriched in SFAs and monounsaturated fatty acids, especially palmitate (16:0) and oleate (18:1) (Table S1). Surprisingly, PE, PE(P), PS and PI all appeared to be quite insensitive to this oversupply, their overall fatty acid profile remaining similar whatever the diet (Figs S4-S11).

By contrast, PC was the most affected class of PL, but in a very selective organ-specific manner. The liver and muscles were the most affected organs (Figs 2 and 3B), a process that could be more clearly visualized when performing PCA (Fig. 4B,E,F). In these tissues, DB=1 species appeared to be increased (PC 32:1 and PC 34:1 in the liver; PC 34:1 in muscles) at the expense of PUFA-containing species (PC 36:4 and PC 38:4 in the liver; PC 36:4 and PC 38:6 in muscles; Fig. 2). Notably, the same tendency was observed for PE and PS, but to lower levels and below the level of significance (Figs S5, S6, S9 and S10). Interestingly, PCA also revealed that noticeable modifications of the fatty acid pattern could be observed in the spleen, the pancreas and the lung, whereas the cardiovascular system and the brain remained largely unaffected by the HFHF diet (Fig. 4B,E,F).

Tandem mass spectrometry (MS/MS) experiments provided more information about the fatty acid composition of PC species (for a representative example, see Fig. S2C). As shown in Table 1, a closer look at MS/MS analyses revealed that, in muscles, PC species have the same composition of fatty acids (the same PC subspecies) in CTL and HFHF diets. Furthermore, MS results showed that PC(16:0/18:1) clearly accumulated at the expense of AA [PC(16:0/20:4)]- and DHA [PC(16:0/22:6)]-containing species under the HFHF diet (Fig. 2). This result reflected the fatty acid composition of the HFHF diet, which is not only enriched in 16:0 and 18:1 but also contains lower amounts of linoleic (18:2) and linolenic (18:3) acids, the respective precursors for AA (20:4) and DHA (22:6), than the standard diet.

By contrast, in the liver the situation appeared more complex than expected. Indeed, increased amounts of PC 32:1 and PC 34:1 could be partly accounted for in this organ by the appearance of PC species that were not detected in the liver of rats under the standard diet, namely PC(14:0/18:1) and PC(16:1/18:0) (Table 1). Notably, 14:0, 16:1 and 18:0 are also enriched in the HFHF diet (Table S1). Moreover, decreased amounts of PC 36:4 and PC 38:4 were not only related to a global decrease in AA-containing species, but were also accompanied by the formation of new species, namely PC(18:2/18:2) and PC(18:1/20:3), respectively (Table 1).

To summarize, two main organs appear highly reactive to the selective fatty acid enrichment from the diet, namely the liver and skeletal muscles. Palmitate and oleate preferentially distribute within PC in the form of PC 34:1. Interestingly, this fatty acid redistribution occurred without modifications to the percentage of PC among total PL (Fig. S12). This observation suggests that this modification of the PC fatty acid pattern does not result from an increased synthesis of PC at the expense of other PL species, but rather from a selective distribution of exogenous fatty acids originating from the diet towards this selective PL class. Moreover, a decrease in the amount of PUFA-containing species can also be observed in the same organs. This can be explained, at least for AA-containing species, by decreased amounts of the relevant precursor (namely linoleic acid) in the HFHF diet.

### Lipotoxicity in the liver

**Fig. 5.**
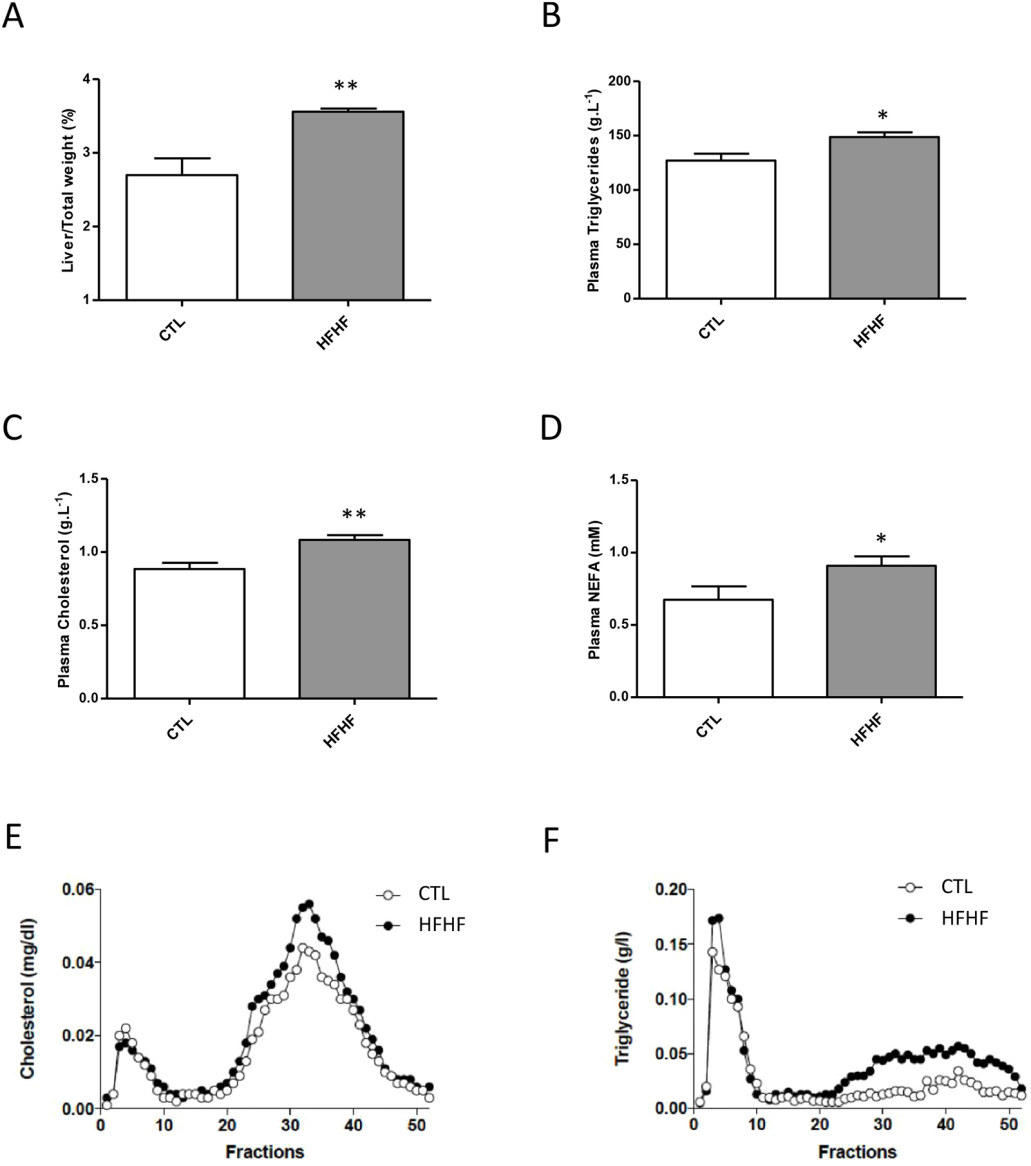
**Effects of the diet on liver weight and circulating lipid profiles.** (A-F) Livers from rats fed either a control (CTL) or HFHF diet for 15 weeks were dissected and weighed; the liver/total weight ratio was determined (A). A total of 15 weeks after the initiation of the different diets, plasma triglycerides (B), cholesterol (C) and NEFA (D) levels were measured after a 3-h fasting period to allow gastric emptying. In parallel, plasma samples were collected and subjected to fractionation by FPLC and cholesterol (E) and triglyceride (F) concentrations in each fraction were measured. See the Materials and Methods section for details. All determinations were performed on six rats from each group. Values are the mean±s.d. Parameters were compared between CTL and HFHF rats using unpaired Student's *t*-test. **P<0.01 and *P<0.05.

Among the various tissues, the liver was clearly one of the main targets of diet-induced fatty acid rearrangements in PL (Figs 2 and 3). This was not a surprising observation, because the liver has a key role in lipid metabolism as the hub of fatty acid synthesis and lipid circulation through lipoprotein synthesis (Nguyen et al., 2008). In the HFHF model (Lozano et al., 2016), alterations in liver function were manifested as early as 2 months following the shift to the enriched diet. The main manifestations at this early time point were the induction of steatosis, with a steatosis score of 1-2 (according to Kleiner et al., 2005), and increased hepatic levels of reactive oxygen species. In the present study, we also showed that 4 months of HFHF resulted in a significant increase in liver weight (Fig. 5A) and in the amounts of circulating lipids, including triglycerides (Fig. 5B), cholesterol (Fig. 5C) and NEFAs (Fig. 5D). Analysis of the lipoprotein profile by fast protein liquid chromatography (FPLC) revealed that HFHF rats displayed increased plasma concentrations of lipoproteins rich in cholesterol [low-density lipoprotein (LDL) and high-density lipoprotein (HDL); Fig. 5E]. Moreover, we also noted an increase in triglyceride levels within chylomicrons/very-low-density lipoprotein (fractions 3-10) and within LDL/HDL (fractions 20-50, Fig. 5F).

As hepatic deposition of neutral lipids is a hallmark of steatosis, we evaluated if such a deposition could be visualized in the HFHF model. With this aim, MS analyses were performed on non-purified lipid extracts from liver samples (Fig. S13). As shown, clear-cut accumulations of triglycerides, diglycerides and free cholesterol could be seen in the liver of HFHF rats (Fig. S13). These findings were confirmed using another analytical method: thin layer chromatography of hepatic neutral lipid fractions (Fig. S14). These observations confirmed previous data from Lozano et al. (2016), showing a significant increase in triglycerides in the liver as early as 2 months following induction of the HFHF diet, a situation that was maintained after 8 months. Interestingly, selective triglyceride (TG) and diglyceride (DG) species appeared to accumulate in the liver under HFHF, the main ones being TG(52:2), TG(54:5), DG(34:1) and DG(36:2) (Fig. S13). Complementary MS/MS analyses (S.K. and T.F., unpublished data) showed that these lipids corresponded to 16:0- and 18:1-containing species, namely TG(16:0/18:1/18:1), TG(16:0/18:1/20:4), DG(16:0/18:1) and DG(18:1/18:1), reflecting the fatty acid composition of the HFHF diet.

### Lipotoxicity and muscle function

Apart from the liver, muscles were the most affected tissues in terms of their sensitivity to fatty acid rearrangements within PLs (Figs 2 and 3). Interestingly, however, in contrast to the liver, this redistribution was not paralleled by the deposition of neutral lipids (triglycerides, diglycerides and cholesterol; Figs S13 and S14). Fatty acid rearrangements corresponded to a decrease in the amount of DHA-containing PC species (namely PC 38:6), a lipid species that was exquisitely enriched in these tissues (Fig. 2 and Table 1). Notably, the same observations were made in both the fast-twitch extensor digitorum longus (EDL) and slow-twitch (soleus) types of muscle (Fig. 2).

**Fig. 6.**
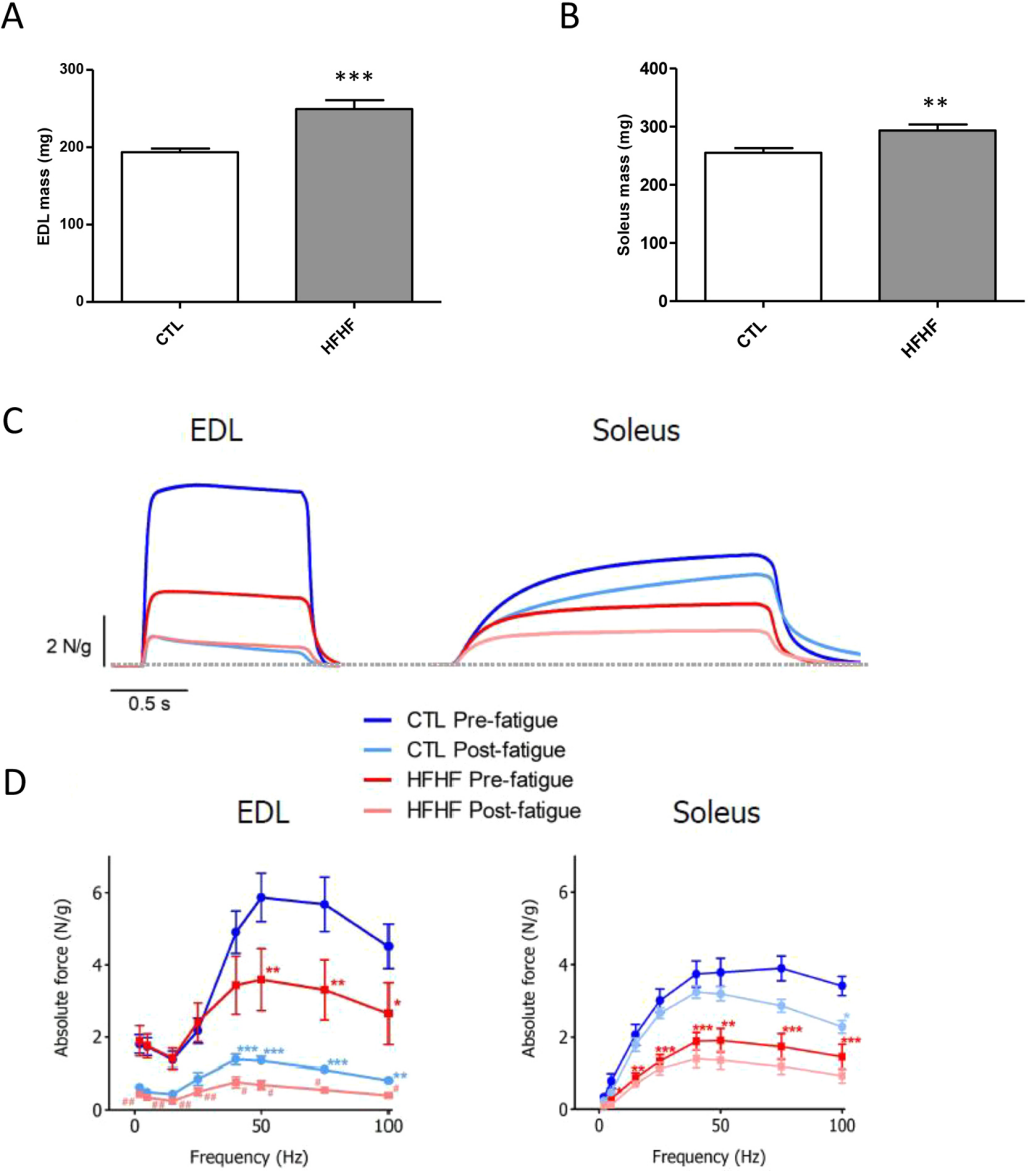
**Effects of the diet on the function of skeletal muscles.** (A,B) A total of 15 weeks after the initiation of the different diets (CTL or HFHF), the EDL (A) and soleus (B) muscles were dissected and their weight was determined for comparison between CTL and HFHF rats (*n*=11). Values are means±s.e.m. Parameters were compared between CTL and HFHF rats using unpaired Student's *t*-test. (C,D) Effects of the HFHF diet on tetanus amplitude and fatigue of EDL and soleus muscle. Examples of tetanus responses to electrical field stimulation at 100 Hz for EDL (left) and soleus (right) before and after a fatigue protocol in CTL (blue traces) and HFHF rats (red traces) (C). Force-frequency relationships for the same types of muscle (D). Values are means±s.e.m. Statistical tests were performed using one-way analysis of variance and a Dunnett's multiple comparison as post-test; ***P<0.001, **P<0.01 and *P<0.05.

Obesity can cause a decline in the contractile function of skeletal muscle. Isolated muscle preparations show that obesity often leads to a decrease in the force produced per muscle cross-sectional area and power produced per muscle mass (Tallis et al., 2018). Therefore, we further investigated the effects of the HFHF diet on the functional features of both fast-twitch and slow-twitch skeletal muscles. In this regard, the fast-twitch EDL muscles and the slow-twitch soleus muscles were isolated from rats on both diets. A first observation was that the absolute mass of these skeletal muscles was increased under the HFHF diet (Fig. 6A,B). In a next step, soleus and EDL muscles were stimulated with field electrodes to measure force characteristics in two different states: before fatigue (pre-fatigue) and immediately after a fatigue protocol (post-fatigue) (Fig. 6C,D).

Fig. 6C displays examples of tetanus force responses, relative to muscle mass, in EDL and soleus muscles from control CTL and HFHF rats in the two pre-fatigue and post-fatigue stimulation conditions. In these examples, force was classically found to be weaker in CTL soleus muscle than in EDL CTL muscles in pre-fatigue conditions (mean values EDL 4.5±0.6 N/g, soleus 3.4±0.3 N/g). Tetanic force was found to be impaired by the HFHF diet in EDL muscles and was also reduced, but to lesser effect, in soleus muscles (Fig. 6C). Force-frequency curves (Fig. 6D) were analyzed for both EDL and soleus muscles. The HFHF diet (red curves) resulted in a decreased muscle tetanic force compared with CTL, starting at stimulation frequencies greater than 40-50 Hz (at 100 Hz, EDL 2.6±0.8 N/g in the HFHF group versus 4.5±0.6 N/g in the CTL group; soleus 1.5±0.3 N/g in the HFHF group versus 3.4±0.3 N/g in the CTL group). These findings indicate that a HFHF diet impairs contractile force in both fast-twitch and slow-twitch muscles.

Moreover, a significant impairment of tetanic force was observed after fatigue protocols. At 100 Hz, in CTL EDL muscle, the fatigue protocol led to an 82% decrease (from 4.5±0.6 to 0.8±0.1 N/g) of tetanic force and this decrease reached 85% in HFHF EDL (from 2.6±0.8 to 0.4±0.1 N/g). The same effect, to a lesser extent, was recorded in soleus: 32% (from 3.4±0.3 to 2.3±0.2 N/g) in CTL and 39% (from 1.5±0.3 to 0.9±0.2 N/g) in HFHF.

To conclude, adaptations occurring in response to the HFHF diet result in a general muscle force loss consistent with that observed in humans (Sayer et al., 2005). These findings would imply that the HFHF diet induces a drastic decrease in the tetanus force, whatever the type of muscle, without significantly changing the behavior towards fatigue.

### Lipotoxicity and the cardiovascular system

In contrast to the liver and skeletal muscles, the cardiovascular system appeared to be quite protected from diet-induced fatty acid redistribution within PLs (Figs 2 and 3). However, as there is much evidence to suggest a relationship between obesity and cardiovascular disease (CVD) in humans, even if the degree and the duration of obesity appears to affect the severity of CVD (Ortega Francisco et al., 2016), we decided to further evaluate the effects of the HFHF diet on the cardiovascular system.

**Fig. 7.**
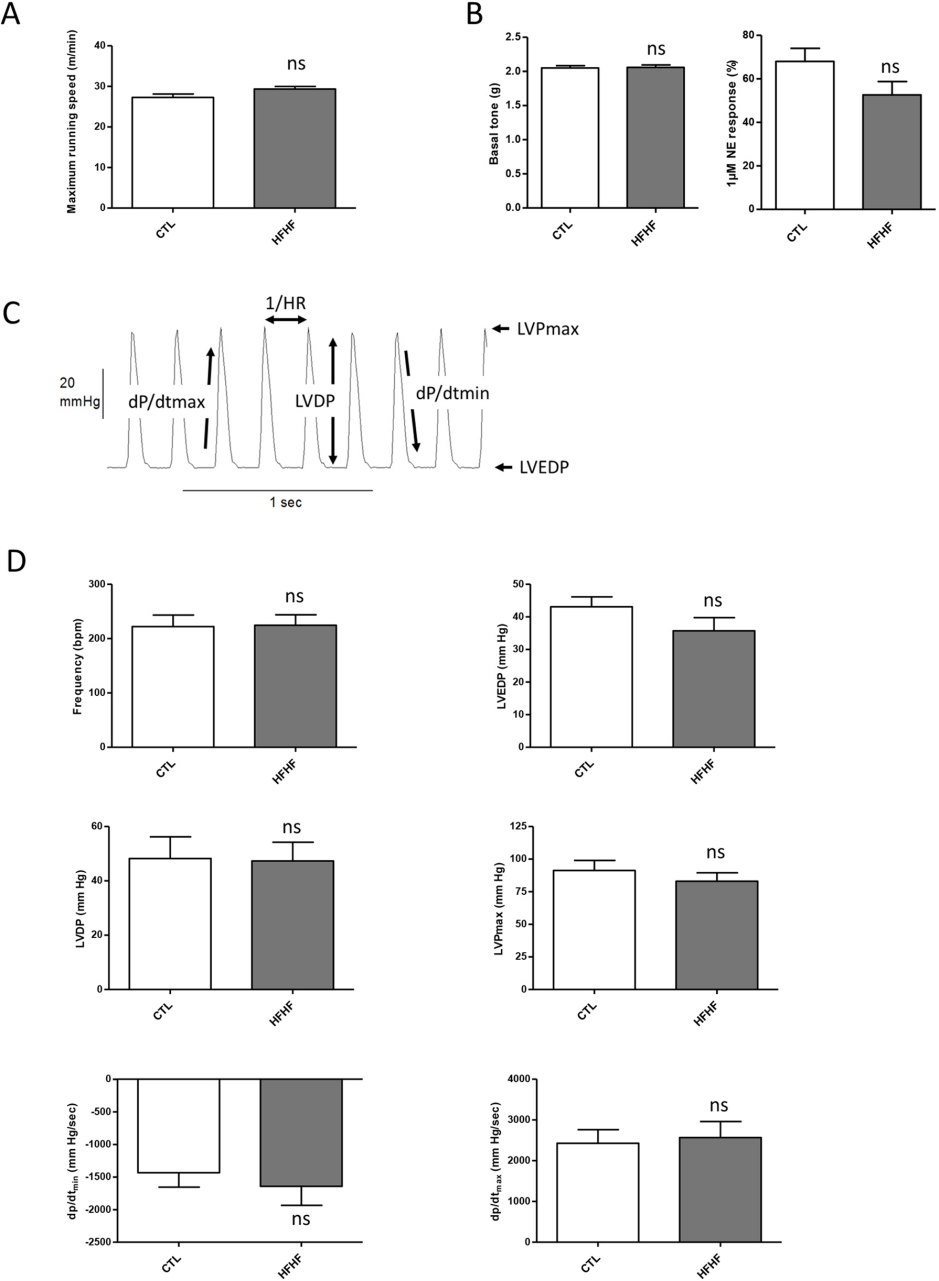
**Effects of the diet on the cardiovascular system.** (A) The maximum running speed was determined 15 weeks after the initiation of the different diets: CTL (*n*=7) or HFHF (*n*=8). (B) At the same time point, the basal tone and the induced contraction was measured on rat aorta rings. Aorta rings obtained from four CTL and five HFHF rats were mounted between a fixed clamp and incubated in Krebs solution to determine the basal tone (left panel); norepinephrine (NE; 1 µM) was added to the same aorta rings to evoke the sustained contractile response (right panel). (C,D) The pressure developed by the contractile left ventricle of the animals was also determined using a Langendorff set-up. Rat hearts from either CTL (*n*=11) or HFHF (*n*=12) groups were submitted to the protocol illustrated in Fig. S15. Parameters recorded during the whole protocol are illustrated in C. The results obtained during the pre-ischemic period are presented in D as means±s.e.m. Pre-ischemic parameters were compared between CTL and HFHF rats using unpaired Student's *t*-test; ns, non-significant. bpm, beats/min; dP/dt_max_, maximal contraction velocity; dP/dt_min_, maximal relaxation velocity; LVDP, left-ventricular developed pressure; LVEDP, left-ventricular end-diastolic pressure; LVPmax, maximum left-ventricular pressure.

First, a maximal exercise test was used as an indicator of the cardiorespiratory capacity of rats. Interestingly, the maximum running speeds (MRS) of the control and HFHF group were not significantly different post-diet, with values of 27.8±0.83 m/min and 29.4±0.61 m/min, respectively (Fig. 7A). Therefore, HFHF clearly did not impair the global functional capacity of the animals.

Obese subjects with insulin resistance and hypertension have abnormal aortic elastic function, which might predispose them to the development of left ventricular dysfunction (Robinson et al., 2008). In this context, we compared the basal tone on CTL and HFHF rat aorta, but we observed no difference between the two types of aorta rings (Fig. 7B). Norepinephrine-induced vasoconstriction was also similar on CTL and HFHF rat aorta rings (Fig. 7B).

Direct cardiac structural abnormalities and alterations in ventricular function have been shown to occur in severely obese patients and in a process that might predispose them to heart failure. More specifically, left ventricular hypertrophy in severe obesity (either eccentric or concentric) is frequently observed and the direct implication of metabolic disturbance, including lipotoxicity, has been suggested (Ortega Francisco et al., 2016).

The left ventricular balloon system allows for real-time monitoring of the pressure developed by the contractile left ventricle of hearts mounted in a Langendorff set-up. Rat hearts from either CTL (*n*=11) or HFHF (*n*=12) groups were submitted to the protocol illustrated in Fig. S15. Parameters recorded during the pre-ischemic period (Fig. 7C) are illustrated in Fig. 7D, which shows that no significant difference was found between the two groups whatever the parameter under consideration. In other words, the contractile behavior of rat hearts was unaffected by the feeding diet imposed on rats during the 15 weeks preceding the experiment.

When the hearts from both groups were submitted to global ischemia (Fig. S16), contractile performances rapidly decreased until developed pressure completely vanished. Again, no significant difference was observed between CTL and HFHF rats, whatever the recorded parameter. When perfusion was restored, as illustrated in Fig. S16, cardiac performance rapidly recovered. It seems that there is a tendency of hearts from CTL rats to recover better than HFHF hearts during reperfusion; however, this tendency only reaches statistical significance for left ventricular diastolic pressure at 1 min and 5 min following reperfusion onset, with all other values remaining non-significantly different between the two groups (Fig. S16).

To conclude, on the HFHF diet under the conditions employed, the cardiovascular system seemed to be functionally protected during the early onset of metabolic syndrome.

## DISCUSSION

It has been known for a long time that the fatty acid composition of the diet can influence the PL signature in various tissues (for a review, see Gimenez et al., 2011). An important conclusion from the present study, however, is that not all organs are equal in this redistribution. Indeed, in the HFHF rat model, most organs were protected from fatty acid rearrangements, at least during early onset of metabolic syndrome, with the liver and skeletal muscles being the preferred targets (Figs 2 and 3). A second important conclusion of the present study is that fatty acids from the diet can distribute within PLs in a very selective way: PC appears to be the preferred sink for this distribution.

In the liver, the redistribution of fatty acids was paralleled by the deposition of neutral lipids, including diglycerides, triglycerides, cholesterol and steryl esters (Figs S13 and S14). Hepatic deposition of neutral lipids is a hallmark of dyslipidemia in obesity and is thought to promote hepatic insulin resistance associated with non-alcoholic fatty liver disease, which is a major factor in the pathogenesis of type 2 diabetes and metabolic syndrome (Klop et al., 2013; Kumashiro et al., 2011). As observed for PC, the main triglyceride and diglyceride species that accumulated in this organ corresponded to those containing the fatty acids that are specifically enriched in the HFHF diet used in this study: i.e. 16:0 and 18:1 (Table S1), PC(16:0/18:1) (Table 1), TG(16:0/18:1/18:1), TG(16:0/18:1/20:4), DG(16:0/18:1) and DG(18:1/18:1) (Fig. S13). Interestingly, in obese individuals, diglycerides can inhibit insulin signaling by activation of protein kinase C isoforms (Kumashiro et al., 2011). In these patients, as in the HFHF model, hepatic diglycerides composed of 16:0/18:1 and 18:1/18:1 are most abundant and strongly related to insulin resistance (Kumashiro et al., 2011). Accordingly, in the present study, HFHF resulted in a significant increase in the concentrations of circulating glucose (Fig. 1B) and insulin (data not shown). These observations confirm previous data from Lozano et al. (2016), which showed that after 2 months the HOMA2-IR (homeostasis model assessment) values were higher than 2.4 in HFHF rats. These data demonstrated insulin resistance in this model at a very early stage in the onset of metabolic syndrome. To summarize, similar lipid depositions/rearrangements are observed in the livers of obese individuals and HFHF rats and are likely to have similar effects on the initiation of the insulin resistance phenotype.

If the effects of HFHF on the liver were not a surprising observation, as this organ has a key role in lipid metabolism, the fact that skeletal muscles were the second most affected tissues in terms of PC fatty acid rearrangements was less predictable (Fig. 2). Importantly, in contrast to the liver, this redistribution was not accompanied by deposition of neutral lipids in these tissues (Figs S13 and S14).

Obesity is generally associated with changes in muscle quality, as it appears to result in larger muscles of lower quality (i.e. less contractile force per unit of cross-sectional area and lower power output per unit of muscle mass), which have the same absolute force and power output of smaller muscles in lean individuals (Tallis et al., 2018). These same observations were made in the present study, where HFHF resulted in absolute increases in EDL and soleus masses (Fig. 6A,B) but reduced force per muscle mass (Fig. 6C,D). Increase in muscle mass probably compensates for poor muscle quality, at least in the early onset of metabolic syndrome, as manifested by the similar performances of HFHF and CTL rats in the maximal exercise tests (Fig. 7A). If the connection between muscle force and the observed decrease in PUFA-containing PLs remains correlative at this step, knowing the importance of such lipid species in membrane plasticity/elasticity (Antonny et al., 2015; Pinot et al., 2014), additional experiments aimed at studying the intimate relationships between the levels of PUFA-enriched PL, the membrane properties of muscle cells and their ability to stretch/contract will undoubtedly shed new light on these mechanisms.

Finally, the fact that the cardiovascular system was protected from fatty acid rearrangements within PL and remained largely unaffected from a functional point of view was also unexpected. These observations suggest that protective mechanisms underlying dyslipidemia exist to channel excess fatty acids to skeletal muscles rather than to the cardiovascular system. It has been demonstrated that the prognosis of CVD for a patient who recently became obese might be different from another who has been obese for many years (Ortega Francisco et al., 2016). In a pioneering study, Nakajima et al. (1985) demonstrated that alterations in cardiac performance in obese individuals is attributed not only to excess body weight, but also to the duration of obesity. Long-lasting experiences to evaluate the effects of HFHF on the cardiovascular system, both on PL fatty acid signature and overall performance, will undoubtedly help to establish clearer connections between these processes.

To conclude, to our knowledge, this is the first study of its kind to provide an overview of the distribution of fatty acids originating from the diet within PLs in various organs and to assess their functional performance. Further studies aimed at establishing direct links between PL fatty acid composition, relevant membrane properties in targeted cells and organ function, particularly in the later steps of metabolic syndrome, will help us to further understand the effects of lipotoxicity on the progression of the associated diseases/comorbidities in this complex pathology.

## MATERIALS AND METHODS

### Animals

The present study was approved by the Comité d'Ethique et d'Expérimentation Animale (COMETHEA) and the French Ministère de l'Enseignement Supérieur, de la Recherche et de l'Innovation (authorization no. 2016071215184098). The protocols were designed according to the Guiding Principles in the Care and Use of Animals approved by the Council of the American Physiological Society and were in adherence with the Guide for the Care and Use of Laboratory Animals published by the US National Institutes of Health (NIH Publication no. 85-23, revised 1996) and according to the European Parliament Directive 2010/63 EU.

We recapitulated the model developed by Lozano et al. (2016). A total of 28 male 8-week-old Wistar rats (275-299 g), supplied by Envigo (Gannat, France), were housed in a temperature-controlled room, in a 12 h light/dark cycle environment with *ad libitum* access to water and food (random fed conditions). After 1 week of acclimatization, the rats were randomly divided into two groups of 14 rats each. The first group had free access to a standard diet (CTL) from MUCEDOLA (Settimo Milanese, Italy) with the macronutrient composition: 3.0% fat, 18.5% protein, 46% carbohydrate, 6% fiber and 7% ash (minerals). The second group ‘high fat high fructose’ (HFHF) received a purified laboratory hypercaloric rodent diet ‘WESTERN RD’ (SDS, Special Diets Services, Saint Gratien, France) containing 21.4% fat, 17.5% protein, 50% carbohydrate, 3.5% fiber, 4.1% ash and an additional 25% fructose (Sigma-Aldrich, Saint-Louis, MO, USA) in water. Fatty acid distribution within the fat fraction of both diets is shown in Table S1. In ‘WESTERN RD’, among the fatty acids, the mono-unsaturated fatty acid oleate and the saturated fatty acid palmitate were the most common and were found in equal amounts, comprising 60% of the total fatty acids within the fat fraction. During the longitudinal observation, body weight was measured each week and plasma glucose, triglyceride and cholesterol levels were determined every 2 weeks at the same time of day (14:00). A final experiment was performed 15 weeks after initiation of different diets. To reduce the heterogeneity between animals, food was removed 3 h before blood glucose measurement and blood sampling for the assessment of insulin, cholesterol, triglyceride and NEFA levels.

All rats were sacrificed 16 weeks after starting administration of each diet for further lipidomic profiling and *ex vivo* experiments.

### Biochemical plasma analysis

Blood glucose levels were measured using an automatic glucose monitor (One Touch vita, LifeScan Inc., Milpitas, CA, USA). Plasma triglyceride and total cholesterol concentrations were determined using commercially available colorimetric kits (Sobioda, Montbonnot-Saint-Martin, France). Plasma-free fatty acids were quantified by a colorimetric NEFA kit (Wako Chemicals, Osaka, Japan). For lipoprotein characterization, plasma samples were collected and subjected to fractionation by FPLC (ÄKTA pure chromatography system, GE Healthcare Life Sciences, Chicago, IL, USA). Cholesterol and triglyceride concentrations in each fraction were measured using commercially available colorimetric kits (Sobioda).

### Lipid extraction, PL purification and MS analyses

After rats were anesthetized, the organs were quickly removed and put on ice. These organs were cut into small pieces (1-2 mm^3^) and dipped in liquid nitrogen. The frozen pieces were introduced into cryotubes before immersion in liquid nitrogen for storage at −80°C.

Lipids were extracted from individual samples, according to the following procedure. Each frozen sample was first submitted to three rounds of grinding using a Precellys Evolution homogenizer (Bertin Technologies, Montigny-le-Bretonneux, France) and resuspended in 1 ml water before transfer to glass tubes containing 500 μl of glass beads (diameter 0.3-0.4 mm; Sigma-Aldrich). Lipids were extracted using chloroform/methanol (2:1, v/v) and shaking with an orbital shaker (IKAH VXR basic VibraxH, Sigma-Aldrich) at 1500 rpm for at least 1 h, as described elsewhere (Kadri et al., 2018). The final organic phase was evaporated and dissolved in 100 μl dichloromethane for purification of PL on a silica column (Bond ELUT-SI, Agilent Technologies, Santa Clara, CA, USA). Lipid samples were loaded on the top of the column. Non-polar lipids were eluted by the addition of 2 ml dichloromethane and glycolipids with 3 ml acetone. PLs were then eluted using 2 ml chloroform/methanol/H_2_O (50:45:5, v/v/v).

PL analysis by MS was performed using a direct infusion of purified lipid extracts on a Synapt G2 HDMS (Waters Corporation, Milford, MA, USA) equipped with an electrospray ionization source (ESI). The mass spectrum of each sample was acquired in the profile mode over 1 min. The scan range for PL analysis was 500-1200 m/z. PS, PI, PE and PE(P) species were analyzed in negative-ion mode after the addition of 0.1% (v/v) triethylamine (Fig. S1A). PC species were analyzed in positive-ion mode after the addition of 0.1% (v/v) formic acid, as already described (Kadri et al., 2018) (Fig. S2A). Identification of the various PL species was based on their exact mass using the ALEX pipeline (Husen et al., 2013), and on MS/MS fragmentation for structural confirmation of the polar head (PL class) and determination of the fatty acid composition. MS/MS experiments of PI, PS, PE and PE(P) were performed by collision-induced dissociation in the negative-ion mode. Examples of MS/MS spectra obtained in the negative-ion mode for some PLs are presented in Fig. S1B-D; the spectra show characteristic fragment ions allowing the identification of PL structures. MS/MS experiments in negative-ion mode also allowed the identification of fatty acid chains in PC species, as shown in Fig. S2C. An MS/MS spectrum in positive-ion mode led to the identification of the polar head of PC class (a characteristic and prominent fragment for all PC species with 184 m/z, as shown in Fig. S2B).

Complementary experiments were conducted on muscle and liver samples using non-purified lipid extracts in positive-ion mode with a scan range from 300 to 1200 m/z to detect neutral lipid species like cholesterol, diglycerides and triglycerides (Fig. S13). The structure of these compounds was confirmed based on the exact mass using the ALEX pipeline (Husen et al., 2013) and on MS/MS fragmentation of the main species. In addition, to evaluate the percentage contribution of PC, PE, PS and PI to total PL in the affected organs (liver and muscle) in response to diet, a mixture of internal standards of the corresponding PL was added and quantification was performed based on full MS spectrum in positive and negative-ion modes (for further details see Fig. S12). All spectra were recorded with the help of MassLynx software (Version 4.1, Waters). Data processing of MS/MS spectra in this work was carried out with Biovia Draw 19.1^©^ and MassLynx^©^ software, with the help of Lipid Maps Lipidomics Gateway^®^ (https://www.lipidmaps.org/).

### Exercise

To determine the effect of diet on the functional capacity of rats, a maximal exercise test was used (Carneiro-Júnior et al., 2013). In this test, rats from the CTL (*n*=7) and HFHF (*n*=8) groups ran on a treadmill (Exer3/6 Treadmill, Columbus Instruments, Columbus, OH, USA) for 5 min at 13 m/min at a grade of 10 degrees, and the speed was increased by 3.6 m/min every 2 min until the animals were exhausted. At this time, the speed measured was their maximum running speed (MRS). The week before the first MRS test, a treadmill habituation session was performed.

### Muscle preparation and contraction measurement

EDL and soleus muscles were carefully dissected with tendons intact on both ends and then vertically tied between a fixed hook at the bottom of the water-jacketed 100 ml chamber (EmkaBATH2, Emka Electronique, Noyant-la-Gravoyère, France) and the force transducer (MLTF500ST, ADInstruments, Dunedin, New Zealand) by means of cotton threads. Before experiments, muscles were maintained for 10 min in the 25°C physiological solution chamber under oxygenated conditions (95% O_2_ and 5% CO_2_). The physiological solution (Krebs solution) contained 120 mmol/l NaCl, 5 mmol/l KCl, 2 mmol/l CaCl_2_, 1 mmol/l MgCl_2_, 1 mmol/l NaH_2_PO_4_, 25 mmol/l NaHCO_3_ and 11 mmol/l glucose (pH 7.4). The isometric tension was recorded by means of the transducer through a module (PowerLab 2/26, ADInstruments) driven by the LabChart7 software (ADInstruments). Electrical external field stimulation was delivered through a constant current stimulator (STM4, Bionic Instruments, Grenoble, France) and a pair of platinum electrodes (Radnoti, Terenure, Ireland) flanking both sides of the isolated muscle. Optimum stimulation conditions and muscle length were established in the course of preliminary experiments. In our device set-up, supramaximal stimulation amplitude proved to be 200 mA for a duration of 1 ms, the optimum length achieved for a resting pre-tension of 2 g.

The force-frequency relation was achieved by increasing step-by-step the frequency of iterative stimulation current pulses (supramaximal amplitude and duration indicated above) as follows: 2, 5, 15, 25, 40, 50, 75 and 100 Hz. For EDL, each sequence of multiple pulses was applied for 1 s followed by a relaxing period of 1 s; for soleus, pulse sequences were applied for 2 s followed by a 1 s relaxing period. The absolute force was measured as the amplitude at the end of the pulse and normalized to the muscle weight (in N/g of muscle). The assessment of muscle fatigue was achieved by performing force-frequency relation protocol before (pre-fatigue) and 30 s after (post-fatigue) a fatigue protocol consisting of 30 successive sets of stimulations at 100 Hz.

### Contraction measurement on isolated aortic rings

The thoracic aorta of rats was removed and placed into Krebs solution containing 120 mM NaCl, 4.7 mM KCl, 2.5 mM CaCl_2_, 1.2 mM MgCl_2_, 1.2 mM KH_2_PO_4_, 15 mM NaHCO_3_ and 11.1 mM D-glucose (pH 7.4). After separation of connective tissues, the thoracic segment of the aorta was cut into rings of 3 mm in length. Rat aorta rings were mounted between a fixed clamp at the base of a water-jacketed 5 ml organ bath containing oxygenated (95% O_2_ and 5% CO_2_) Krebs solution and an IT1-25 isometric force transducer (Emka Technologies, Paris, France) (Fortner et al., 2001; Vandebrouck et al., 2006). All experiments were performed at 37°C. A basal tension of 2 g was applied in all experiments. After 1 h, tissues were rinsed three times in Krebs solution and the basal tone was monitored and adjusted to the range 400-1000 mg (Watson et al., 1998). To evoke the sustained contractile response, 1 µM norepinephrine was used.

### Langendorff perfusion analyses

A left ventricular balloon system allows for real-time monitoring of the pressure developed by the contractile left ventricle of hearts mounted in a Langendorff set-up. To achieve this goal, CTL or HFHF rats were anesthetized by intraperitoneal injection of sodium pentobarbital (60 mg/kg). The heart was quickly removed and the ascending aorta was connected according to the Langendorff technique (Bell et al., 2011) and a 0.06 ml latex balloon (VK 73-3479) was inserted in the left ventricle.

The balloon was connected to a pressure transducer, which was linked to the data acquisition system (PowerLab 425, ADInstruments). Hemodynamic and functional parameters were recorded on a personal computer using LabChart software (ADInstruments). Hearts were allowed to stabilize for 30 min while perfused with standard Krebs solution. Functional parameters were assessed before, during and after a 30 min long ischemic insult. In the preischemic period, the ventricular pressure was monitored under standard perfusion conditions for 10 min (Fig. S15). Then, ischemia was induced by complete cessation of coronary flow for 30 min (global ischemia). After this time, reperfusion was initiated by re-establishing coronary flow and cardiac parameters were recorded and analyzed for the next 30 min.

### Statistical analysis

*P*-values were calculated either by two-tailed Student's *t-*tests or ANOVA and completed by adequate post-tests, as indicated in the corresponding figure legends. These analyses were performed using the GraphPad Prism 5 software. Multivariate PCAs were performed using Past 4.01 software (https://folk.uio.no/ohammer/past/).

## Supplementary Material

Supplementary information
